# Extreme temperatures and cardiovascular mortality: assessing effect modification by subgroups in Ganzhou, China

**DOI:** 10.1080/16549716.2021.1965305

**Published:** 2021-09-06

**Authors:** Wei Zhang, Gang Du, Liang Xiong, Tingting Liu, Zuobing Zheng, Qiong Yuan, Jiahui Yang, Yangna Wu, Rongfei Zhu, Gonghua Hu

**Affiliations:** a Key Laboratory of Prevention and Treatment of Cardiovascular and Cerebrovascular Diseases of Ministry of Education, Gannan Medical University, Ganzhou, China; b Department of Occupational Health and Occupational Medicine, School of Public Health and Health Management, Gannan Medical University, Ganzhou, Jiangxi, China; c Ganzhou Center For Disease Control And Prevention, Ganzhou, Jiangxi, China

**Keywords:** Extreme temperatures, cardiovascular disease, distributed lag non-linear model, mortality

## Abstract

**Background:**

Many people die from cardiovascular diseases each year, and extreme temperatures are regarded as a risk factor for cardiovascular deaths. However, the relationship between temperature and cardiovascular deaths varies in different regions because of population density, demographic inequality, and economic situation, and the evidence in Ganzhou, China is limited and inconclusive.

**Objective:**

This study aimed to assess extreme temperature-related cardiovascular mortality and identify the potential vulnerable people.

**Methods:**

After controlling other meteorological measures, air pollution, seasonality, relative humidity, day of the week, and public holidays, we examined temperature-related cardiovascular mortality along 21 lag days by Poisson in Ganzhou, China.

**Results:**

A J-shaped relationship was observed between mean temperature and cardiovascular mortality. Extremely low temperatures substantially increased the relative risks (RR) of cardiovascular mortality. The effect of cold temperature was delayed by 2–6 days and persisted for 4–10 days. However, the risk of cardiovascular mortality related to extremely high temperatures was not significant (*p* > 0.05). Subgroup analysis indicated that extremely low temperatures had a stronger association with cardiovascular mortality in people with cerebrovascular diseases (RR: 1.282, 95% confidence interval [CI]: 1.020–1.611), males (RR: 1.492, 95% CI: 1.175–1.896), married people (RR: 1.590, 95% CI: 1.224–2.064), and people above the age of 65 years (RR: 1.641, 95% CI: 1.106–2.434) than in people with ischemic heart disease, females, unmarried people, and the elderly (≥65 years old), respectively.

**Conclusions:**

The type of cardiovascular disease, sex, age, and marital status modified the effects of extremely low temperatures on the risk of cardiovascular mortality. These findings may help local governments to establish warning systems and precautionary measures to reduce temperature-related cardiovascular mortality.

## Background

Cardiovascular diseases (CVDs) are the leading cause of deaths worldwide. Approximately 17.9 million people die from CVDs every year, and over 75% of CVD-related death’s occur in low- and middle-income compared with high-income countries [1,2]. Extreme temperatures can increase the risk of excess mortality [3–5]. Similar to cardiovascular risk factors, such as smoking, heavy drinking, being underweight or obese, and physical inactivity [6,7], increasing attention have been paid to the effects of temperature on CVDs by public health authorities. People with underlying CVDs are susceptible to extreme temperatures [8,9].

Cardiovascular mortality and ambient temperature have a nonlinear U-, V-, or J-shaped relationship [10,11]. According to this relationship, both high and low temperatures can increase the risk of excess deaths [9,12,13]. However, the relationship between ambient temperature and cardiovascular mortality varies in different climatic regions [14,15]. For instance, people living in temperate areas are more sensitive to high temperatures than those in the subtropical areas. Moreover, this relationship is evidently contradictory even in different cities with the same climate [16–18]. This discrepancy can be attributed to distinct longitudes or latitudes, population density, demographic inequality, and economic situation [9,15,19,20]. However, current data are insufficient to extrapolate this relationship to other areas.

To date, the relationship between temperature and cardiovascular mortality in Ganzhou City remains unclear. Moreover, only a few studies have evaluated the correlation between cause-specific CVDs and individual characteristics, which is a better measure for identifying vulnerable people [13,21,22]. A better understanding of the temperature–mortality relationship can facilitate the establishment of local early warning systems and precautionary measures to reduce the adverse health effects of extreme temperatures. In the present study, we implemented stratified analyses based on the types of CVDs, gender, age group, and marital status for identifying subpopulations that are susceptible to the effects of extreme temperatures.

## Methods

### Study setting

**Figure 1.**
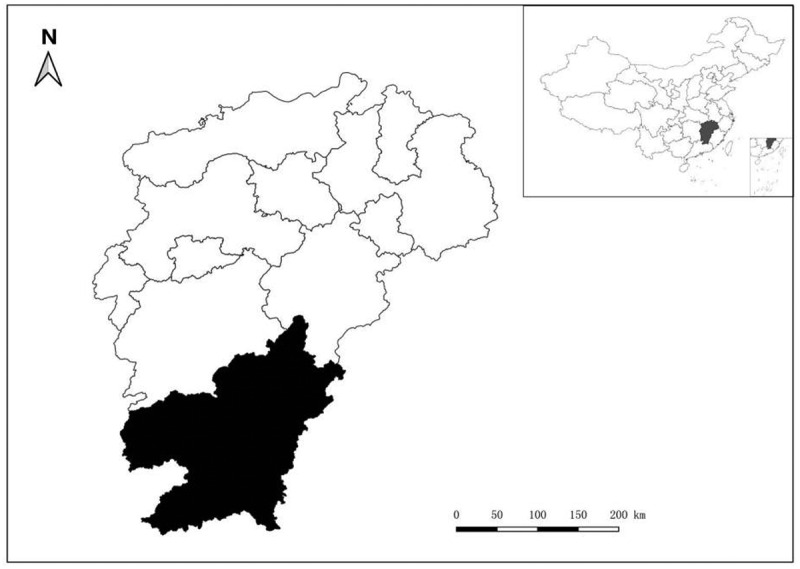
The city (in black) into this research

Ganzhou City (N 24°29ʹ–27°09ʹ, E 113°54ʹ–116°38ʹ, Figure 1), which is located east of China, is the largest city with the largest population in Jiangxi Province. By the end of 2019, the total registered population of the entire city was 9.8307 million. This city with a subtropical monsoon climate is surrounded by mountains, and it is one of the key non-ferrous metal bases in China.

### Data collection

The daily meteorological and air pollution data from 2015 to 2019 were downloaded from the China Meteorological Data Sharing Service System, the authoritative sharing service platform for meteorological data resources operated by China Meteorological Administration. The data included maximum, mean, and minimum temperatures, relative humidity, and air pressure, which were obtained from a single monitoring station in Ganxian District (N 114°02ʹ, E 25°85ʹ). Air pollution, including the daily 24-hour average concentrations of particulate matter with an aerodynamic diameter less than 10 (PM_10_) and 2.5 µm (PM_2.5_), sulfur dioxide (SO_2_), nitrogen dioxide (NO_2_), carbon monoxide (CO), and 8-hour moving average ozone (O_3_) concentration at several stations in the center of Ganzhou. Considering the lack of meteorological data from Ganzhou City, the temperature records from the nearest county, which is approximately 19 kilometers away from the air pollution monitoring stations, with a similar latitude and altitude were used. For evaluating city-wide temperature effects on mortality, time series model based on one monitoring station’s temperature is equal to spatiotemporal model utilizing spatial temperatures [23]. The effects of daily ambient temperature on cardiovascular deaths were adjusted by considering air pollutants as the confounding factors in the model.

Daily counts of cardiovascular deaths from 1 January 2015 to 31 December 2019 were obtained from the China Information System of Death Register and Ganzhou Center for Disease Control and Prevention. The causes of death were coded according to the International Classification of Diseases, 10th Revision (ICD-10): CVD (ICD-10: I00-I99), ischemic heart disease (IHD; I20-I25), and cerebrovascular disease (CBD; I60-I69). Moreover, cardiovascular deaths were stratified into groups based on cause-specific CVD (IHD, HPD, and CBD), gender, marital status (married and unmarried, including never married, divorced, or widowed), and age (<65 years and ≥65 years).

### Statistical analysis

Many studies demonstrated that the distributed lag non-linear model (DLNM) can accurately evaluate the relationship between daily temperature and health outcomes [11,15,19]. Moreover, the daily death toll follows the Poisson distribution [24]. Accordingly, DLNM combined with Poisson generalized linear regression was used to examine the relationship between daily temperature and cardiovascular mortality.

The general model for analysis is as follows:

$$Y_{t}\setminus Poisson(u_{t})$$

_(1)_ $$Log(u_{t})=\alpha +cb(Temp_{t,l}^{\;},df)+ns(time_{t},n/year*5)+DOW_{t}+COVs$$

where *μ_t_* denotes the expected counts of cardiovascular death on lag day *t, α* is the intercept, *cb* expresses the ‘cross-basis’ function, *Temp* represents the expected daily mean temperature, *l* is the lag day for temperature, *ns* is the natural cubic spline function, *time_t_* describes long-term trend and seasonality, *n* is the degree of freedom (df) of time trend, *DOW_t_* is an indicator variable for the day of the week (DOW), *COVs* are the potential confounding factors, including relative humidity, air pressure, and concentrations of air pollution (PM_2.5_, PM_10_, SO_2_, NO_2_, CO, and O_3_), and the degrees of freedom is 3; dichotomous variables indicating the day of public holiday were based on previous studies [17,21,25].

The ‘cross-basis’ function was used to examine the two-dimensional relationship between the dimensions of temperature and lag days. In ‘cross-basis’ function, the cubic b-spline function was utilized for daily mean temperature, and the polynomial function was adopted for the lag structure. Daily mean temperature has been used to appraise and accurately predict temperature related-cardiovascular mortality [8,21,26]. In the present study, 21 days were considered as the time window to examine the overall effects. The model uses df = 5 in the mean temperature [21,23]. The minimum value of the Akaike information criterion (AIC) for the quasi-Poisson models was used to select the optimum degrees of freedom for time trend and lag structure [25]. In the final model, we used df = 4 for time trend and df = 4 for lag.

In the present study, 1% and 99% daily temperatures were defined as extremely low and extremely high temperatures, respectively. The daily median temperature (21.9℃) was used as the reference temperature to evaluate all effects. To observe the effects of extreme temperature change, we evaluated the relative risks (RR) for cardiovascular mortality at the 5th and 95th percentiles of temperature, respectively. Moreover, RRs with 95% confidence interval (CI) were calculated for cardiovascular mortality at extremely low and high temperatures along specific lag days. Three-dimensional diagrams were drawn to observe the effects between temperature indicators and cardiovascular mortality at the overall lag days. Two-dimensional plots were produced to accurately show the relationship of daily temperatures with RRs and lag days with RRs. Lag effects along lags of 0, 0–7, 0–14, and 0–21 days were also calculated to estimate the cumulative effects of extreme temperatures.

### Sensitivity analysis

The main findings of this study were validated by analyzing the sensitivity of model by changing the df from 6 to 9 per year for time trend. We also evaluated the effects of other daily temperature indicators (maximum and minimum temperatures) instead of mean temperature in the basic model. Moreover, we used an alternative maximum lag of 14 or 28 days. All data were analyzed using the ‘dlnm’ package in R (version 4.0.3).

## Results

### Descriptive results

A total of 103,645 cardiovascular mortality counts in Ganzhou City were recorded during the study period. Meteorological, air pollution, and cardiovascular mortality data are summarized in Table 1. The average minimum temperature was 17.16 ± 7.70°C (mean±SD), the mean of daily average temperature was 18.5 ± 7.7°C, the average maximum temperature was 20.39 ± 8.00°C, and relative humidity was 76.39%±12.02%. Among the cardiovascular deaths, 29,897 patients died from IHD, while 45,350 patients died from CBD. Among these patients, 56,767 were males, 74,440 were married, and 81,924 were 65 years or older. The average age of patients who died from CVDs was 74.2 ± 13.4 years with a range of 1–114 years. The daily counts of cardiovascular mortality evidently fluctuated with the seasons and peaked in winter.

**Table 1.** Summary statistics of meteorological data and air pollution (mean (SD)), mortality (mean (%)) from cause-specific cardiovascular diseases and individual characteristics, in Ganzhou during

Variables	**Mean**		**Min**	**P25**	**P50**	**P75**	**Max**
Meteorological data							
MinT(°C)	17.2	(7.7)	−3.0	10.4	18.6	24.3	30.0
MeanT (°C)	20.4	(8.0)	1.0	13.6	21.9	27.5	33.0
MaxT(°C)	25.0	(8.8)	1.7	18.0	26.7	32.6	39.0
Relative humidity (%)	76.4	(12.0)	36.0	67.0	77.0	86.0	99.0
Air pressure (hPa)	998.6	(7.9)	980.7	992.1	998.3	1004.7	1024.4
Air pollution							
PM2.5 (ug/m^3^)	40.7	(20.3)	7.0	27.0	36.0	51.0	180.0
PM10 (ug/m^3^)	63.4	(32.6)	12.0	40.3	55.0	79.0	250.0
SO2 (ug/m^3^)	22.4	(13.2)	3.0	13.0	19.0	28.0	110.0
NO2 (ug/m^3^)	23.8	(12.4)	8.0	15.0	20.0	29.0	84.0
CO (mg/m^3^)	1.3	(0.3)	0.6	1.1	1.3	1.5	3.0
O3 (ug/m^3^)	59.4	(25.5)	5.0	41.0	57.0	75.0	167.0
Cause-specific mortality							
CVD	57	(100.0)	14	44	54	67	142
HPD	11	(19.3)	0	8	10	13	33
IHD	16	(28.1)	3	12	16	20	46
CBD	25	(43.9)	1	19	24	37	72
Gender							
Males	31	(54.4)	7	24	30	37	87
Females	26	(45.6)	4	19	24	31	68
Marital status							
Unmarried	16	(28.1)	1	12	15	19	52
Married	41	(71.9)	9	31	39	48	100
Age							
<65 years	12	(21.1)	1	9	11	15	39
≥65 years	45	(78.9)	8	34	43	53	120

2015 to 2019.

Note: MeanT: daily mean temperature; P25, 25th percentile; P50, 50th percentile; P75, 75th percentile;

### Exploratory results

**Figure 2.**
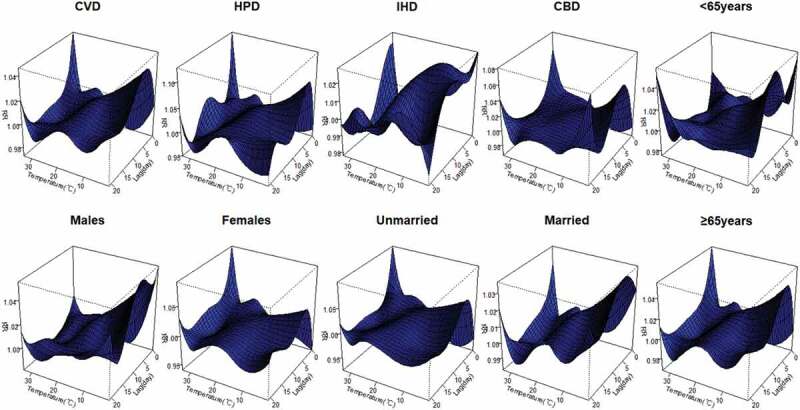
Three-dimensional graph of the relative risks of cardiovascular mortality by daily mean temperature (°C) days and lag days, during 2015–2019. The relative risks for mortality of types of CVD and individual characteristics. The relative risks used eight of degree of freedom for time trend and daily mean temperature for temperature indicator. The reference temperature was the median temperature during the study period

The three-dimensional plots show the non-linear relationship of daily average temperature and RRs along the lag of 21 days. The highest effect of high temperatures was mainly recorded on current lag 0 day (Figure 2).

Fig.S2 shows the hot effect of daily mean temperature along the 21 lag days, but no statistical significance was observed. Figure S1 presents the cold effect of daily mean temperature along the 21 lag days. For CVD mortality, the cold effect was delayed by 3 days, persisted for 8 days, and peaked on lag 5–6 days. For HPD mortality, the cold effect was observed after 2 days, lasted for 6 days, and peaked on lag 4 days (RR: 1.067, 95% CI: 1.030–1.105). However, for IHD mortality, exposure to extremely low temperatures did not increase the mortality. For CBD mortality, the cold effect was delayed by 4 days, lasted for 1 week, and peaked on lag 6 days (RR: 1.041, 95% CI: 1.017–1.065).

In terms of individual characteristics, extremely low temperatures substantially increased the risk of death among males, but this effect was not observed in females. For unmarried people, the effects of cold temperatures were observed after 4 days, lasted for 5 days, and peaked at lag 5 days (RR: 1.043, 95% CI: 1.014–1.074). For married people, the effects of cold temperatures were observed after 3 days, lasted for 10 days, and peaked at lag 5–7 days. The cold effect was observed 4 days earlier among the elderly than among patients below the age of 65 years, lasted for 1 week, and peaked at lag 5 days (RR: 1.037, 95% CI: 1.015–1.060; Table S1).

**Figure 3.**
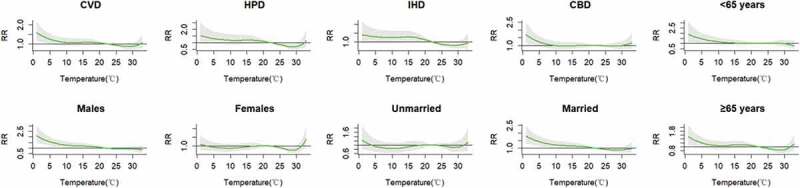
The relationship between mean temperature and the mortality of types of CVDs and individual characteristics and over lag 0–21 days, during 2015–2019. The green lines are the maximum likelihood estimate of relative risks, and the gray regions are 95% CIs. The solid line marks RR at 1

**Figure 4.**
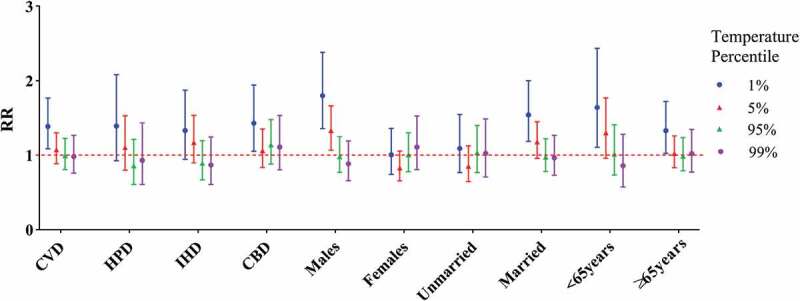
RRs showing the effect modification by various mortality subgroups at the 1st, 5th, 95th, and 99th temperature percentiles

The cumulative RRs of cause-specific cardiovascular mortality and individual characteristics over 21 days were estimated (Figure 3). A J-shaped relationship was observed between daily mean temperature and cardiovascular mortality. Low temperatures increased the RRs for certain subpopulations, whereas high temperatures did not show harmful effects. The cumulative cold effects on cause-specific cardiovascular mortality and individual characteristics at lag 0, 0–7, 0–14, and 0–21 days are presented in Table 2. The cumulative hot effect was not statistically significant (data not shown). For CVD, CBD, males, married people, and people below the age of 65 years, the highest cumulative cold effect was reached at lag 0–21 days. For HPD, IHD, and the elderly, the highest cumulative cold effect was reached at lag 0–14 days. The highest cumulative cold effect was observed among males (RR: 1.798, 95% CI: 1.358–2.381). In Figure 4, the effect of extreme temperatures and the 95th and 5th percentile of temperature modification can be observed.

**Table 2.** The cumulative cold effects on lag 0, 0–7, 0–14, 0–21 days from cause-specific cardiovascular disease and individual characteristics

	lag 0	lag 0–7	lag 0–14	lag 0–21
Cause-specific mortality				
CVD	0.992 (0.927–1.061)	1.195 (1.039–1.376)*	1.341 (1.108–1.622)*	1.384 (1.087–1.764)*
HPD	0.967 (0.864–1.082)	1.357 (1.072–1.716)*	1.402 (1.019–1.928)*	1.389 (0.926–2.083)
IHD	1.019 (0.927–1.121)	1.165 (0.955–1.421)	1.324 (1.012–1.732)*	1.332 (0.947–1.873)
CBD	0.973 (0.894–1.060)	1.160 (0.972–1.385)	1.327 (1.043–1.687)*	1.428 (1.051–1.940)*
Gender				
Males	1.028 (0.951–1.111)	1.347 (1.145–1.585)*	1.671 (1.341–2.082)*	1.798 (1.358–2.381)*
Females	0.948 (0.871–1.032)	1.034 (0.867–1.233)	1.022 (0.805–1.298)	1.005 (0.743–1.361)
Marital status				
Unmarried	0.937 (0.850–1.032)	1.115 (0.911–1.365)	1.148 (0.873–1.510)	1.091 (0.769–1.546)
Married	1.011 (0.940–1.088)	1.223 (1.050–1.423)*	1.421 (1.157–1.745)*	1.539 (1.185–2.000)*
Age				
<65 years	1.027 (0.921–1.145)	1.193 (0.949–1.499)	1.567 (1.152–2.132)*	1.641 (1.106–2.434)*
≥65 years	0.983 (0.914–1.057)	1.197 (1.029–1.391)*	1.289 (1.051–1.582)*	1.328 (1.024–1.722)*

*p < 0.05.

### Sensitivity analyses

Three sensitive analyses were performed to check the robustness of our main findings. First, we changed df (6–9 per year) for time trend, in which the effects of the daily mean temperature on cardiovascular mortality were insensitive. Second, other temperature indicators (maximum and minimum temperatures) were used to replace daily mean temperature, and the results show that the relationship between temperature and cardiovascular mortality was similar. Third, we changed the maximum lag days from 14 to 28, in which no substantial change was incurred (Fig. S2).

## Discussion

We examined the relationship between daily mean temperature and cardiovascular mortality in Ganzhou from 2015 to 2019. These variables showed a J-shaped nonlinear association. Low temperatures caused temperature-related cardiovascular mortality. Cold effect was delayed by 2–4 days and persisted for 5–12 days. By contrast, the effect of high temperatures was not significant. Subpopulation analysis revealed that people with CBD, males, married people, and people aged <65 years were more vulnerable to extremely low temperatures.

Almost every subtype of CVD hospitalizations and mortality peak during the winter, and most of the attributable cardiovascular deaths are caused by low temperature rather than high temperature [10,27]. Similar results were obtained in the present study.

High temperatures exacerbate the risks of cardiovascular mortality, particularly in vulnerable groups [24,28]. Hot effects are immediate and are usually associated with short lags [17,29,30]. However, this study did not show any association between high temperatures and cardiovascular mortality in Ganzhou, thus supporting the findings in subtropical cities, such as Shanghai [21] and Hong Kong [29], in China. Moreover, Ma et al. [9] reported that when the latitude was lower than 34.2°, the hot effect increased the mortality by 2.23% per 1°C increase. This phenomenon occurred possibly because people living in warm areas are usually more acclimated to high temperatures and are highly sensitive to cold weather [21,29]. With global warming, heat waves and hot weather are likely to increase in intensity and frequency [31]. In Ganzhou, cardiovascular mortality caused by high temperatures should be the focus of future works.

Cold effects lasted for 10–15 days similar to our findings in Ganzhou [4,16]. However, considering the discrepancies in latitudes, socioeconomic statuses, and demographic profiles, the effects of low temperatures on CVD in different cities are widely divergent. In Beijing, China, low temperatures substantially increased the risk of cause-specific cardiovascular CVD mortality, especially HPD mortality [21]. In Germany, the risk of CVD and CBD mortality caused by low temperatures was high, whereas the risk of IHD mortality caused by low temperatures was not significant [8]. Our results indicated that people with CBD are more susceptible to extremely low temperatures than to high temperatures. Lin et al. [32] found that extremely low temperatures are associated with the risk of emergency room visits for patients with CBD and the risk of outpatient visits for patients with IHD.

In the present study, the risk of cardiovascular mortality among males exposed to extremely low temperatures was higher than that among females, consistent with the results of a previous study [17,19]. Males are commonly engaged in outdoor work and activities, thus increasing their exposure to extreme temperatures [20]. However, other investigations showed that females have stronger risks of cardiovascular mortality than males [16,33]. This discrepancy in various locations can be attributed to geographical context, ratio of women and men, and local climate between the sexes [14,33].

The elderly with pre-existing health conditions are highly vulnerable to hot and/or cold temperatures, and this trend is evident during the winter [34–36]. The effects of extreme temperatures vary among different age groups [29,37]. A systematic review and meta-analysis showed that a percentage change per 1°C decrease in temperature below a threshold in winter months increases cardiovascular mortality among the elderly (3.44%, 95% CI: 3.10–3.78), and a percentage change per 1℃ increase in temperature above a threshold increases cardiovascular mortality in this age group (1.66%, 95% CI: 1.19–2.14) [38]. The elderly (age ≥65 years) are more sensitive to extremely low temperatures than people below the age of 65 years [16,23]. However, other studies reported the opposite trend, which was similar to our results [39,40]. This discrepancy might be attributed to differences in cultural and health-related factors [41]. Our findings were obtained possibly because the elderly are likely to spend more time indoors [8], thus reducing their exposure to extreme temperatures. The small sample size of this study might have also influenced the results.

To date, no study has reported the effects of temperature on CVD in a population with different marital statuses. The mortality associated with hypothermia from high to low is as follows: widow > married > single > divorced [42]. In Yuxi city of China, the effect of low temperature on the total non-accidental mortality of married people is high [43]. In Hong Kong, the mortality among married people is higher than that among unmarried people [44]. Furthermore, in the diurnal temperature range of low, high, extremely low, and extremely high temperature, the mortality of married people increased [45]. Therefore, married people are more sensitive to extreme temperature than unmarried people. A recent study indicated that unmarried people are at higher risk of dying from CVD than married people [46]. However, in the present study, this risk was aggravated by exposure to extreme temperatures, and this result was similar to above-mentioned studies. Current evidence on the effect modification of temperature by marital status is extremely limited. The differences in temperature-related mortality among different marital status needs further investigation.

The mechanism between temperature and cardiovascular mortality remains to be determined. The possible mechanisms are as follows: first, exposure to cold air increases plasma fibrinogen; second, factor VII clotting activity and hemoconcentration increase the risk of vascular thromboses [47,48] and trigger cumulative increases in the levels of inflammatory blood markers (such as C-reactive protein and interleukin-6) [27]. Furthermore, under hot weather, hemoconcentration caused by sweating and perspiration increases blood viscosity and the risk of thrombosis [49]. Both low and high temperatures can increase the blood pressure and heart rate [49–51].

## Study limitations

In the present study, we could not adjust for seasonal influenza. Influenza is usually prevalent during winter, and this condition could confound the effect of cold temperature. Upper respiratory tract infection may also have adverse effects on cardiovascular system during cold season [37]. Furthermore, only one geographic location was considered. Thus, extrapolating the findings to other areas will be difficult. Moreover, accurate temperature exposures for individuals were not available, because people often take measures (e.g, using air conditioners or staying indoors) to reduce the influence of extreme temperatures on themselves.

## Conclusions

People with CBD, males, married people, and people aged <65 years were more vulnerable to extremely low temperatures than to extremely high temperatures. Human adaptation to high ambient temperatures is likely in Ganzhou. These findings may be useful for local governments to establish warning systems and formulate policies to reduce temperature-related mortality.

## Paper context

The non-linear relationship between extreme temperatures and cardiovascular mortality had been widely studied worldwide. Current data cannot be directly extrapolated to other unknown regions because of the divergence of population density and economic situation. This study explored the effect modification in extreme temperatures in terms of cause-specific CVD, gender, marital status, and age to identify vulnerable people. Early warning systems should be established to reduce adverse health effects of extreme temperatures.
